# Chiasmata and the kinetochore component Dam1 are crucial for elimination of erroneous chromosome attachments and centromere oscillation at meiosis I

**DOI:** 10.1098/rsob.200308

**Published:** 2021-02-03

**Authors:** Misuzu Wakiya, Eriko Nishi, Shinnosuke Kawai, Kohei Yamada, Kazuhiro Katsumata, Ami Hirayasu, Yuta Itabashi, Ayumu Yamamoto

**Affiliations:** ^1^ Graduate School of Integrated Science and Technology, Shizuoka University, 836 Ohya, Suruga-ku, Shizuoka 422-8529, Japan; ^2^ Department of Chemistry, Faculty of Science, Shizuoka University, 836 Ohya, Suruga-ku, Shizuoka 422-8529, Japan

**Keywords:** meiosis, kinetochore, sister chromatids, fission yeast, chiasma, chromosome segregation

## Abstract

Establishment of proper chromosome attachments to the spindle requires elimination of erroneous attachments, but the mechanism of this process is not fully understood. During meiosis I, sister chromatids attach to the same spindle pole (mono-oriented attachment), whereas homologous chromosomes attach to opposite poles (bi-oriented attachment), resulting in homologous chromosome segregation. Here, we show that chiasmata that link homologous chromosomes and kinetochore component Dam1 are crucial for elimination of erroneous attachments and oscillation of centromeres between the spindle poles at meiosis I in fission yeast. In chiasma-forming cells, Mad2 and Aurora B kinase, which provides time for attachment correction and destabilizes erroneous attachments, respectively, caused elimination of bi-oriented attachments of sister chromatids, whereas in chiasma-lacking cells, they caused elimination of mono-oriented attachments. In chiasma-forming cells, in addition, homologous centromere oscillation was coordinated. Furthermore, Dam1 contributed to attachment elimination in both chiasma-forming and chiasma-lacking cells, and drove centromere oscillation. These results demonstrate that chiasmata alter attachment correction patterns by enabling error correction factors to eliminate bi-oriented attachment of sister chromatids, and suggest that Dam1 induces elimination of erroneous attachments. The coincidental contribution of chiasmata and Dam1 to centromere oscillation also suggests a potential link between centromere oscillation and attachment elimination.

## Graphical abstract

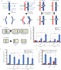

## 1. Introduction

**Figure 1.**
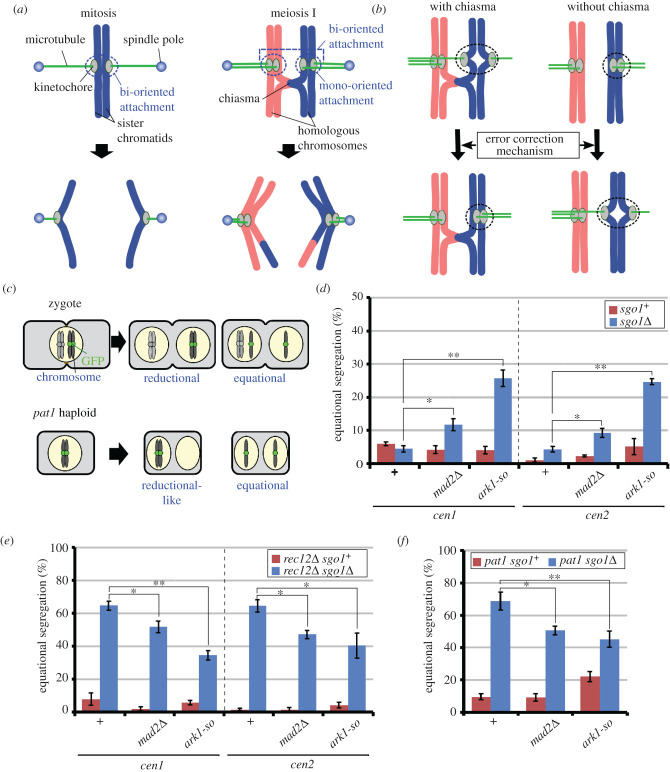
The effects of error correction impairment on sister chromatid segregation at meiosis I. (*a*) Organization, spindle attachment and segregation of chromosomes in mitosis and meiosis I are shown. (*b*) Hypothesis regarding the role of the error correction mechanism in spindle attachment of sister chromatids in the presence and absence of chiasmata. Dotted circles, attachments corrected by the error correction mechanism. (*c*) Segregation patterns of GFP-visualized centromeres of sister chromatids in zygotes and *pat1* haploid cells. (*d*,*e*) Equational segregation frequencies at meiosis I in *rec12^+^* (*d*) and *rec12Δ* cells (*e*). (*f*) Equational segregation of chromosome 2 at meiosis I in *pat1* haploid cells. The *pat1* haploid cells were forced to enter meiosis by activation of the mating pheromone signalling pathway and subsequent inactivation of Pat1 kinase at the restrictive temperature, both of which are required for induction of reductional-like segregation of sister chromatids in haploid cells [1]. In (*d*–*f*), chromosome segregation was analysed in greater than 50 cells, and bars show averages of more than three independent experiments. *cen1*, chromosome 1; *cen2*, chromosome 2; +, no other mutations. Dotted lines in (*d*,*e*) distinguish results for *cen1* and *cen2.* Error bars show standard errors. *p < 0.05; **p < 0.005 (Student's *t*-test). Statistical analysis of error correction-proficient (+) and error correction-defective (*mad2Δ* and *ark1-so*) *sgo1Δ* cells is shown.

Faithful segregation of chromosomes during cell division is essential for genome integrity. During division of a somatic cell (mitosis), duplicated chromosomes (sister chromatids) attach to opposite spindle poles (bi-oriented attachment) via microtubules (MTs) and segregate from each other (equational segregation). Such a division generates two genetically identical daughter cells (figure 1*a*, mitosis) [2]. By contrast, during the first of two consecutive divisions of a germ cell (meiosis), homologous chromosomes attach to opposite poles and segregate, leading to generation of gametes containing half the original number of chromosomes (figure 1*a*, meiosis I).

Proper attachments are established through a dynamic process [3–6]. Chromosomes attach to the spindle randomly via kinetochores, which are protein complexes that assemble at centromeres [7–9]. Kinetochores initially attach to spindle MTs at their lateral side, and subsequently at their ends. Recent studies showed that Ndc80 and the Dam1/DASH complexes (Ska complex in vertebrates) form end-on attachments and couple MT disassembly to centromere movement. Because of the random nature of initial attachment, sister chromatids or homologous chromosomes often erroneously attach to the same pole (mono-oriented attachment) in mitosis or meiosis I, respectively. These erroneous mono-oriented attachments are unstable and are ultimately eliminated; the chromosomes then repeat the attachment and detachment processes until they attach to the spindle properly. In addition, during establishment of attachment, centromeres continuously move back and forth between the spindle poles by forces generated by MT disassembly at the kinetochores [4–6,10]. However, it remains unclear how erroneous attachments are selectively eliminated during oscillation of centromeres.

It is widely believed that proper attachment is established through tension [11,12]. During mitosis, bi-oriented sister chromatids are pulled towards opposite poles, and the pulling forces generate tension because the sisters are held together by a protein complex called cohesin [13]. In the current model, this tension causes stabilization of bi-oriented attachment, whereas tensionless mono-oriented attachment is unstable and subject to elimination. Likewise, at meiosis I, because the homologous chromosomes are linked by recombination products called chiasmata, their bi-oriented attachment generates tension, which is thought to stabilize the attachments [2,11,12].

Several factors have been identified as components of the mechanism that corrects erroneous attachments. The spindle assembly checkpoint (SAC) factors inhibit the onset of anaphase, providing time for correction of erroneous attachments (e.g. [14]), and Aurora B kinase destabilizes erroneous attachments by phosphorylating kinetochore components including Ndc80 and Dam1 [3,15–17]. Consistently, impairment of the SAC pathway or Aurora B kinase increases the frequency of erroneous attachments in both mitosis and meiosis I [18–26]. The enrichment of Aurora B kinase at the inner centromere region, beneath the outer kinetochore, suggests that tension-dependent spatial separation of the outer kinetochores from the inner centromere impedes Aurora-dependent outer kinetochore phosphorylation, thereby stabilizing the attachments [3,19,27].

Although tension-dependent stabilization is currently the widely accepted model for attachment selection, it cannot easily account for attachment selection at meiosis. At meiosis I, homologous chromosomes are attached to opposite poles, while sister chromatids are attached to the same pole. Mono-oriented attachment of sister chromatids is also crucial for homologous chromosome segregation. In the fission yeast *Schizosaccharomyces pombe*, sister centromeres are often bi-oriented and undergo transient splitting [28]. Although these attachments are supposed to generate tension, they are eventually eliminated. In addition, in mouse oocytes, the establishment of correct attachments does not correlate with tension generated at the kinetochores; moreover, tension does not seem to cause spatial separation of the kinetochores from the Aurora-enriched region [20]. Thus, attachment selection at meiosis I is still enigmatic.

At meiosis I, chiasmata are crucial for bi-oriented attachment of homologous chromosomes. However, we previously reported that in *S. pombe*, chiasmata also contribute to mono-oriented attachment of sister chromatids by preventing their bi-oriented attachment [28]. It remains unclear how chiasmata prevent bi-oriented attachment of sister chromatids. Here, we examined chiasma-dependent prevention of bi-oriented attachment in greater detail and found that chiasmata enabled error correction factors to eliminate bi-oriented attachment of sister chromatids and coordinated homologous centromere oscillation. In addition, we found that the kinetochore protein Dam1 contributed to attachment correction and was essential for centromere oscillation. These findings demonstrate the importance of chromosome organization and kinetochore activity in elimination of erroneous attachments and reveal a potential link between centromere oscillation and attachment elimination.

## 2. Results

### 2.1. Chiasmata change the effects of the error correction mechanism on sister chromatid segregation

We previously reported that sister chromatids frequently attach to opposite spindle poles and that chiasmata prevent these attachments [28]. We hypothesized that chiasmata change the attachment types that are eliminated by the error correction mechanism such that, in the presence of chiasmata, the mechanism selectively eliminates bi-oriented attachment of sister chromatids, thereby retaining mono-oriented attachment in contrast with the situation in mitosis (figure 1*b*, with chiasmata). If so, impairment of the error correction mechanism should increase the frequency of bi-oriented attachment in chiasma-forming cells due to defective elimination. Conversely, in cells lacking chiasmata, the error correction mechanism would selectively eliminate mono-oriented attachment of sister chromatids, as in mitosis (figure 1*b*, without chiasmata), and error correction impairment should increase their mono-oriented attachment.

To test this idea, we compromised the error correction mechanism and evaluated spindle attachments of sister chromatids in chiasma-forming and chiasma-lacking cells. To compromise the error correction mechanism, we introduced the *mad2Δ* mutation or the *ark1-so* mutation, which represses meiotic expression of the *ark1* gene that encodes fission yeast Aurora B kinase [25]. The *mad2Δ* mutation is thought to reduce the time available for correcting improper attachments by compromising the SAC pathway, whereas the *ark1-so* mutation impairs attachment elimination and prevents SAC activation. To evaluate attachments, we examined sister chromatid segregation by visualizing the centromere-proximal locus of one of the homologues of chromosome 1 (*cen1*) or 2 (*cen2*) using the lacI/lacO recognition system (figure 1*c*) [1,29]. In this analysis, we deleted the *sgo1* gene, which encodes one of two Shugoshin proteins of fission yeast that specifically functions as a centromere cohesion protector during meiosis and prevents separation of sister chromatids attached to opposite poles [28,30–33].

Segregation of sister chromatids to the opposite poles (equational segregation) was very rare in all types of cells in the *sgo1^+^* background (figure 1*d*), confirming that Sgo1 prevents separation of sister chromatids [28,31,32]. By contrast, in the *sgo1Δ* background, although equational segregation was rare in chiasma-forming cells (figure 1*d*), it was very frequent in diploid *rec12Δ* cells [28], which do not form chiasmata because of the lack of Rec12 (an Spo11 homologue in fission yeast), a factor required for the formation of DNA double-strand breaks (figure 1*e*, +) [34]. Frequent equational segregation was not caused by a loss of Rec12 functions, as demonstrated by the observation that equational segregation was also frequent in haploid meiotic cells, which do not form chiasmata (figure 1*c* and *f*, +) [28]. Importantly, in the *sgo1Δ* background, introduction of the *mad2Δ* or *ark1-so* mutation significantly increased the percentages of equational segregation in chiasma-forming diploid cells (figure 1*d*), but decreased the segregation percentages in chiasma-lacking diploid *rec12Δ* (figure 1*e*) or haploid meiotic cells (figure 1*f*). These results indicate that the error correction mechanism decreases bi-oriented attachment of sister chromatids in the presence of chiasmata, but conversely increases bi-oriented attachment (thereby decreasing mono-oriented attachment) in the absence of chiasmata. In our previous study, the equational segregation frequencies of *cen1* were somewhat higher in the *rec12Δ* background [28], although the reason for this is unknown. However, the *mad2Δ* mutation similarly decreased equational segregation, being consistent with our current results.

### 2.2. Chiasmata change the effects of the error correction mechanism on metaphase sister centromere splitting

**Figure 2.**
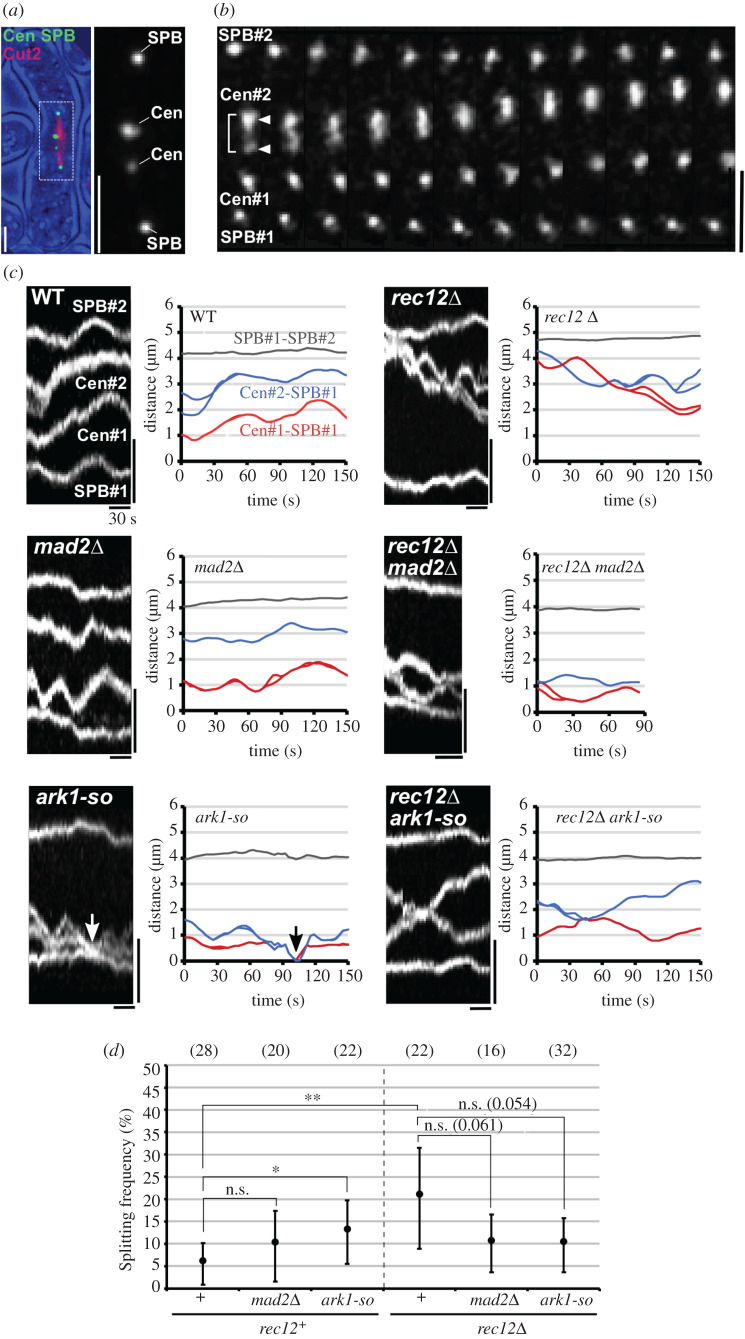
Centromere and SPB dynamics at metaphase I. (*a*) Localization of GFP-visualized centromeres and SPBs and RFP-visualized Cut2 at metaphase I (left), and an enlarged image of centromeres and the SPBs (right), shown by the white box in the left image. Bars, 2 µm. (*b*) Centromere and SPB dynamics at metaphase I in wild-type cells. Maximum projection images of centromeres and SPBs taken every approximately 3 s were arranged from left to right. Arrowheads indicate splitting sister centromeres. Bar, 2 µm. (*c*) Changes in centromere and SPB positions at metaphase I in various cells. Left images show kymograph of centromeres (*cen2*) and SPBs and right graphs show changes in centromere–SPB and SPB–SPB distances. Vertical and horizontal bars of kymographs show 2 µm and 30 s, respectively. Arrows indicate co-localization of centromeres with the SPB. (*d*) Mean observation frequencies of centromere splitting per centromere obtained by the bootstrap method. Graph shows the mean frequencies per centromere. The dotted line distinguishes the results of *rec12^+^* and *rec12Δ* cells. Numbers in parentheses at the top of the graph indicate the number of centromeres examined. Error bars indicate 95% confidence intervals. *p < 0.05; **p < 0.01 (Studentized bootstrap analysis). n.s., not significant. *p*-values > 0.05 and smaller than 0.1 are shown in parentheses.

We next sought to assess bi-oriented attachment of sister chromatids in a more direct manner by examining transient splitting of sister centromeres, a probable outcome of their bi-oriented attachment, which often occurs irrespective of chiasma formation [28]. To precisely evaluate sister centromere splitting during metaphase, we specified metaphase stage by visualizing *S. pombe* securin Cut2, a biochemical marker of the pre-anaphase stage that localizes at the prometa/metaphase spindle (figure 2*a*) [35]. In addition, to follow fine centromere dynamics, we determined three-dimensional positions of *cen2* on the spindle at high temporal resolution by acquiring images of *cen2* and the spindle pole body (SPB; the fungal centrosome) in several different focal planes every approximately 3 s (figure 2*b*). We visualized the SPB using a GFP-tagged SPB component, Sid4 (Sid4-GFP) [36], but left one copy of Sid4 untagged in diploid cells because in our live cell analyses, cells containing two copies of GFP-tagged Sid4 exhibited high frequencies of equational segregation of sister chromatids even in the *sgo1^+^* background (data not shown), which probably resulted from impairment of the function of Sid4 in the regulation of anaphase exit [37,38]. Leaving one copy of untagged Sid4 largely eliminated the segregation abnormalities, as shown by sister chromatid segregation patterns in cells containing one copy of untagged Sid4, which were similar to those observed in cells lacking Sid4 tagging (electronic supplementary material, figure S1).

During metaphase I, centromeres oscillated between the spindle poles in both chiasma-forming wild-type and chiasma-lacking *rec12Δ* diploid cells, as previously reported (figure 2*c*; electronic supplementary material, movies S1 and S2) [28]. In both types of cells, sister centromeres often underwent transient splitting, confirming our previous finding that bi-oriented attachment of sister chromatids occurs irrespective of chiasma formation (figure 2*b,c*) [28]. The frequencies of sister centromere splitting varied (electronic supplementary material, figure S2A), but the mean splitting frequencies per centromere obtained by the bootstrap method were significantly higher in *rec12Δ* cells than in *rec12^+^* cells (the difference was also significant in the usual *t-*test, *p* < 0.01; figure 2*d*, +). In our previous report, the mean splitting frequencies were not significantly different [28]; this discrepancy may be due to the presence of an untagged copy of Sid4 in this study. The elevated frequency of sister centromere splitting in chiasma-lacking cells confirms that chiasmata prevent bi-oriented attachment of sister chromatids.

In *ark1-so rec12^+^* cells, the mean splitting frequency was significantly higher than in *rec12^+^* cells, supporting the notion that bi-oriented attachment of sister chromatids was increased in these cells (figure 2*d*). In addition, although the differences were not significant in other *mad2Δ* or *ark1-so* cells, the mean splitting frequencies tended to be higher in the *rec12^+^* background and lower in the *rec12Δ* background (figure 2*d*; electronic supplementary material, movies S3–S6). These tendencies were consistent with the idea that when the error correction mechanism is impaired, bi-oriented attachment of sister chromatids increases in chiasma-forming cells, but conversely decreases in chiasma-lacking cells. The mechanism driving centromere movement is intact in these mutant cells, as demonstrated by the lack of significant differences in the mean centromere velocities, the mean standard deviations of centromere positions and the plots of the mean square displacement (MSD) of centromeres (electronic supplementary material, figure S2B–D).

**Figure 3.**
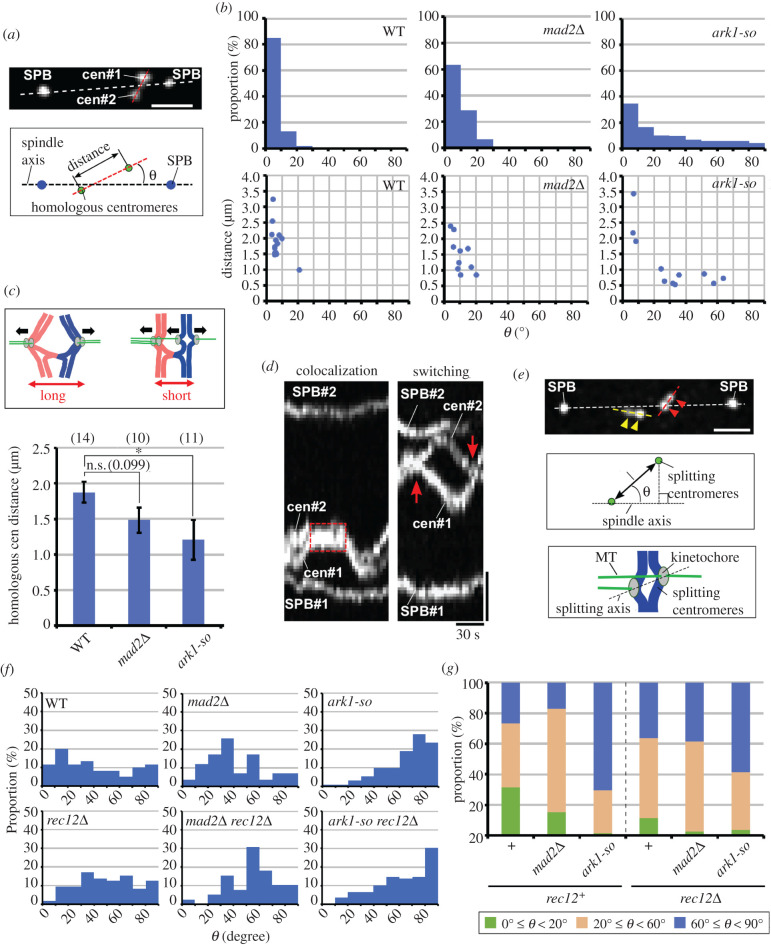
Homologous centromere angles and distances and splitting sister centromere angles. (*a*) Angle of homologous centromeres relative to the spindle and the distance between them. Photo shows the representative image of tilted homologous centromeres. White and red dotted lines in photo show the axes of the spindle and homologous centromeres, respectively. (*b*) Distribution of homologous centromere angles relative to the spindle (upper graphs) and 2D plots of the mean angles and distances of homologous centromeres in each cell (lower graphs). (*c*) Means of the mean distances between homologous centromeres per cell. Bars show means of the mean distances in each cell. Drawings show the predicted relationship between attachments and homologous centromere distance. Black arrows indicate forces exerted on chromosomes. *p < 0.05 (Student's *t*-test). n.s., not significant (number in parenthesis shows *p*-value). Numbers in parentheses at the top of the graph show the numbers of cells examined. Error bars indicate standard errors. (*d*) Colocalization and position switching of homologous centromeres in *ark1-so* cells. Photos show kymographs of homologous centromeres (*cen2*) and the SPBs. The red dotted square in the left photo indicates colocalization of centromeres. Red arrows in the right photo show switching of homologous centromere positions relative to the SPBs. Horizontal bar: 30 s; vertical bar: 1 µm. Note that SPB#2 was out of focus around the end of observation in the right photo. (*e*) Angle of splitting sister centromeres and the distance between them. Image shows splitting sister centromeres (arrowheads) and the SPBs. Dotted lines indicate axes of the spindle (white) and the splitting centromeres (yellow and red). Bar, 1 µm. Upper drawing demonstrates the angle of the splitting centromeres to the spindle axis, ‘*θ*’. Lower drawing demonstrates predicted MT attachments of tilted splitting sister centromeres. (*f*) Distribution of angles of splitting sister centromeres. (*g*) Increased tilting of splitting sister centromeres in *ark1-so* cells. Green bars: 0° ≤ *θ* < 20°; brown bars: 20° ≤ *θ* < 60°; blue bars: 60° ≤ *θ* ≤ 90°. +, no other mutations. Sixty, 58, 132, 169, 22 and 109 splitting sister centromeres were examined in wild-type, *mad2Δ, ark1-so, rec12Δ, mad2Δ rec12Δ* and *ark1-so rec12Δ* cells, respectively.

Additional support for a higher frequency of bi-oriented attachment of sister chromatids in error correction-defective, chiasma-forming cells came from an analysis of homologous centromere positions (figure 3*a*). In wild-type cells, homologous centromeres were positioned mostly nearly parallel to the spindle axis (at angles less than 10° relative to the spindle axis), whereas in *mad2Δ* or *ark1-so* cells, their positions were often tilted and non-parallel (greater than or equal to 10°; figure 3*b*). In addition, homologous centromeres tended to be positioned closer, as demonstrated by a reduction in the mean homologous centromere distances (figure 3*b,c*). Notably, homologous centromere colocalization was observed during 9.2% of total observation time in *ark1-so* cells but was never observed in wild-type cells (figure 3*d*; electronic supplementary material, table S1). These phenotypes indicate a reduction in opposing poleward forces exerted on the homologous centromeres, which likely reflects an increase in the frequency of bi-oriented attachment of sister chromatids. In support of this view, the homologous centromeres occasionally switched their positions relative to the SPB by oscillating independently of each other in *ark1-so* cells (6 switches during 1643 s observation time) (figure 3*d*; electronic supplementary material, table S1), indicating direct exertion of opposite forces on each of the homologous centromeres.

In addition to these findings, analysis of the positions of splitting sister centromeres raised the possibility that in addition to sister chromatids, a single chromatid is often attached to both poles. We noted that splitting sister centromeres were frequently tilted in a variety of directions, sometimes almost perpendicular to the spindle axis in both *rec12^+^* and *rec12Δ* cells (figure 3*e–g*; electronic supplementary material, figure S3), suggesting that spindle attachments of these centromeres are frequently different from simple bi-oriented attachment. Introduction of the *ark1-so* mutation increased the proportion of splitting sister centromeres that tilted towards a perpendicular angle relative to the spindle axis in both *rec12^+^* and *rec12Δ* cells (figure 3*f,g*; electronic supplementary material, figure S3), suggesting that the error correction mechanism contributes to elimination of these attachments irrespective of chiasma formation. Given the correlation between tilted positioning of homologous centromeres and bi-oriented attachment of sister centromeres, the tilted positions of splitting sister centromeres probably reflected bi-oriented attachments of a single centromere (merotelic attachment) together with bi-oriented attachment of sister centromeres (figure 3*e*, bottom drawing).

Considering all these results together, we concluded that chiasmata alter attachment correction patterns by enabling error correction factors to eliminate bi-oriented attachment of sister chromatids.

### 2.3. Chiasmata coordinate homologous centromere oscillation

Because tensionless attachments are thought to be eliminated by the error correction factors, we sought to determine whether chiasmata decrease tension across sister centromeres by measuring inter-sister centromere distances projected on the spindle axis, which probably reflect tension generated by poleward pulling forces (electronic supplementary material, figures S3 and S4). The projected distances in wild-type cells revealed two distinct distribution groups that covered distinct distance ranges, whereas the distances in *rec12Δ* cells revealed a single distribution group (electronic supplementary material, figure S4C). The distribution group that covered the longer distance range in wild-type cells mostly originated from a dataset of a single cell shown in figure 2 (electronic supplementary material, figure S4C, WT w/o). Irrespective of the presence or the absence of this particular dataset, however, the mean projected distance in wild-type cells did not significantly differ from that of *rec12Δ* cells (electronic supplementary material, figure S4D). Therefore, we could not draw a conclusion about whether the tension across sister centromeres is changed by chiasmata.

**Figure 4.**
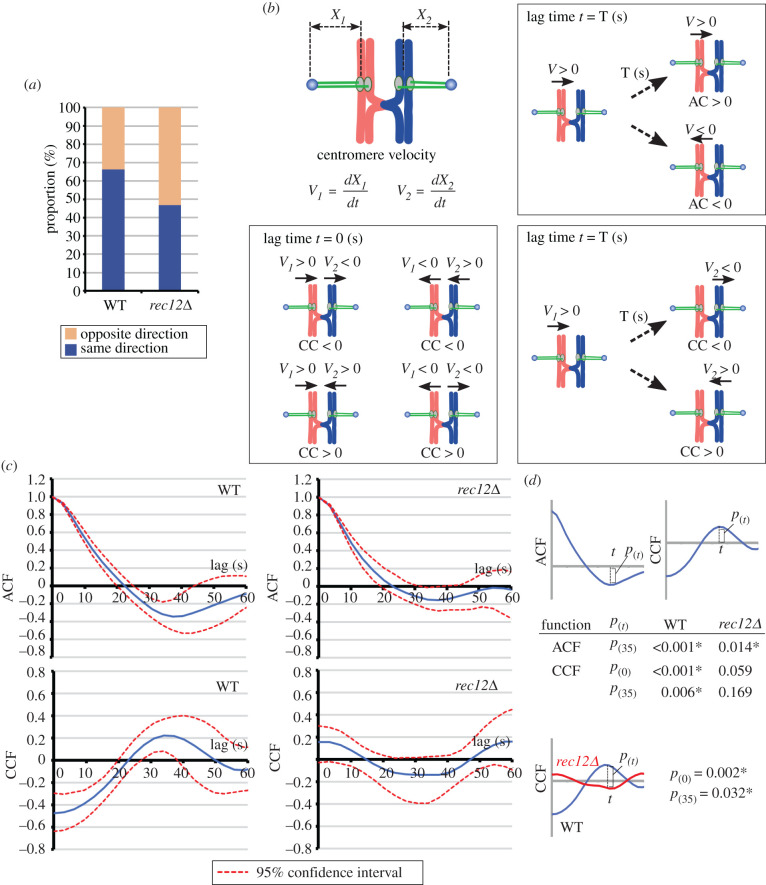
Relationship between homologous centromere movements and correlation analysis of centromere dynamics at metaphase I. (*a*) Movement direction of homologous centromeres. Blue and yellow bars indicate proportions of a pair of homologous centromeres moving in the same and opposite directions, respectively. (*b*) Auto-correlation (AC) and cross-correlation (CC) of centromere velocities. Relationship between correlation values (AC or CC) and centromere movements is shown. Arrows above chromosomes show the direction of centromere movements. (*c*) ACF and CCF of centromere velocities in wild-type and *rec12Δ* cells. Blue lines and red dotted lines, respectively, indicate the mean correlation values and 95% confidence intervals obtained by the bootstrap method. (*d*) *p*-values of ACF and CCF values at the indicated time points. (*e*) *p*-values between CCF values of wild-type and *rec12Δ* cells at the indicated time points. Asterisks in (*d*,*e*) indicate significant differences. The numbers of examined cells and total observation times (shown in parentheses) are the following. Wild-type: 14 cells (1326.8 s); *rec12Δ*: 11 cells (1193.4 s).

Since tension changes could not be assessed in our analyses, we next examined centromere dynamics in more detail to obtain a clue about chiasma-dependent elimination of bi-oriented attachment of sister chromatids. We noted that in chiasma-forming wild-type cells, homologous centromeres tended to move in the same direction and reverse movements in a coordinated manner, whereas this tendency was not apparent in chiasma-lacking *rec12Δ* cells. Indeed, homologous centromeres moved in the same direction during 66.2% and 46.7% of the observation time in wild-type and *rec12Δ* cells, respectively (figure 4*a*); the latter percentage is close to that of random movement. In addition, in *ark1-so* cells, the centromeres often reached the SPB without reversing their movements and co-localized with it (co-localization was observed during 6.4% of observation time in *ark1-so* cells, but never in wild-type cells; figure 2*c*, *ark1-so*, arrows), indicating that centromere reversal is affected by decreased Aurora B activities. These observations suggest a relationship between centromere oscillation and attachment correction.

To confirm chiasma-dependent coordination of homologous centromere oscillation, we characterized centromere movements in a quantitative manner using the correlation function, which is useful for characterizing centromere oscillation (e.g. [39–41]). Specifically, we collected centromere velocities relative to the SPB obtained from all observed cells and calculated the correlation coefficients between velocities separated by various lag times (figure 4*b*). The auto-correlation plot of wild-type cells decreased and reached a negative value; albeit small, the negative value was statistically significant around 35 s (figure 4*c,d*, WT). This indicates that the centromere movements tend to reverse around 35 s in chiasma-forming wild-type cells (figure 4*b*, upper right). The auto-correlation plots of *rec12Δ* cells also exhibited a similar pattern, indicating that centromeres oscillate in chiasma-lacking *rec12Δ* cells in a similar manner (figure 4*c*,*d*, *rec12Δ*). Thus, centromeres oscillate between the two SPBs with a weak but significant periodicity in a chiasma-independent manner.

We next examined the correlation between the velocities of homologous centromeres by cross-correlation analysis. The cross-correlation plot of wild-type cells increased from a negative value and reached a peak at a positive value around 35 s (figure 4*c*), and both the negative and the positive values were statistically significant (figure 4*d*, WT). This shows that homologous centromeres tend to move in the same direction (figure 4*b*, lower left) and reverse their movements every approximately 35 s (figure 4*b*, lower right). Thus, homologous centromere oscillation is coordinated. By contrast, the plot of *rec12Δ* cells differed significantly from the wild-type plot and exhibited no significant correlation at any lag times (figure 4*c*–*e*, *rec12Δ*). This result indicates a lack of coordination in homologous centromere movements. These results demonstrate that chiasmata coordinate homologous centromere oscillation.

We also characterized centromere movements in *ark1-so* cells. The cross-correlation value at 0 s was negative, as in wild-type cells, indicating that homologous centromeres tended to move in the same direction (electronic supplementary material, figure S5A,B). However, the statistical significance of the correlation values of the auto- and cross-correlation plots was lost at around 35 s (electronic supplementary material, figure S5A–C). This observation suggests weakened periodicity of centromere oscillation and is consistent with altered centromere reversal in *ark1-so* cells. By contrast, no such alteration was observed in *mad2Δ* cells, whose major defect is in the SAC-dependent inhibition of precocious anaphase onset (electronic supplementary material, figure S5D–F). The facts that chiasmata coordinate homologous centromere oscillation and that oscillation dynamics are altered in *ark1-so* cells raise the possibility that centromere oscillation is linked to attachment elimination.

### 2.4. Dam1 is required for chiasma-dependent attachment correction

To further explore the relationship between attachment elimination and centromere oscillation, we next searched for kinetochore mutants defective in chiasma-dependent attachment elimination. Among kinetochore proteins, we suspected that Dam1 might contribute to attachment correction and centromere oscillation because Dam1 is an Aurora B substrate and a component of the Dam1/DASH complex that is thought to drive chromosome segregation by coupling MT shortening with kinetochore movements [42–50]; we found that chiasma-dependent attachment elimination was impaired in cells bearing the *dam1* deletion (*dam1Δ*).

*dam1Δ* cells exhibited largely normal spore formation and only a slight decrease in spore viability (electronic supplementary material, figure S6A,B). This observation indicates that meiotic chromosome segregation is not severely impaired in *dam1Δ* cells, being consistent with the fact that Dam1 is not essential for chromosome segregation in mitosis [51]. In addition, both crossover and non-crossover recombination occurred at a wild-type level in *dam1Δ* cells (electronic supplementary material, figure S6*c*), indicating that recombination-dependent chiasma formation is normal in *dam1Δ* cells.

**Figure 5.**
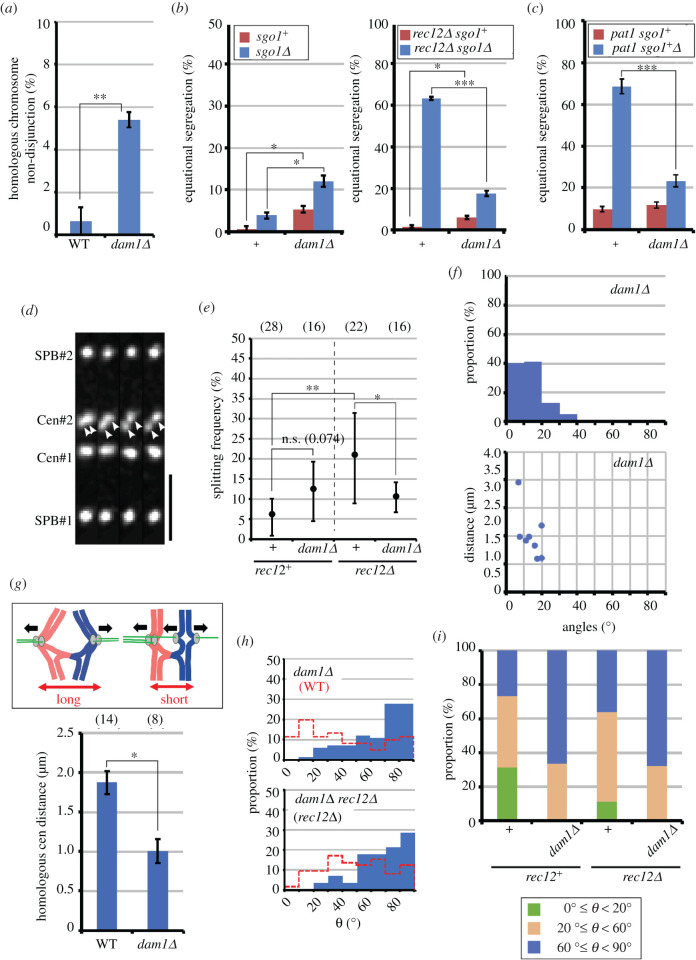
Effects of the *dam1Δ* mutation on chromosome attachment to the spindle at meiosis I. (*a*) Frequencies of non-disjunction of homologous chromosomes (*cen2*). (*b,c*) Equational segregation frequencies of sister chromatids (*cen2*) in diploid (*b*) and *pat1* haploid cells (*c*) at meiosis I. In (*a–c*), chromosome segregation was analysed in more than 50 cells, and bars show averages of more than three independent experiments. Error bars show standard errors. *p < 0.01; **p < 0.005; ***p < 0.0001 (Student's *t*-test). (*d*) Separated sister centromeres in *dam1Δ* cells. Maximum projection images of centromeres and SPBs taken every approximately 3 s were arranged from left to right. Arrowheads show separated sister centromeres. Bar, 2 µm. (*e*) Observation frequencies of centromere splitting obtained by the bootstrap method. Graph shows the mean frequencies per centromere. *p < 0.05; **p < 0.01; n.s., not significant (*p*-value is shown in parentheses). Error bars show 95% confidence intervals. Numbers in parentheses at the top of the graph indicate the numbers of centromeres examined. +, no other mutations. Means of wild-type and *rec12Δ* cells were adopted from figure 2. Dotted line distinguishes results of *rec12^+^* and *rec12Δ* cells. (*f*) Distribution of angles of homologous centromeres relative to the spindle (upper graph), and 2D plots of the mean angles and distances of homologous centromeres in each cell (lower graph). (*g*) Distances between homologous centromeres. Graph shows means of the mean distances in each cell. Drawings show the predicted relationship between attachments and homologous centromere distances. Black arrows indicate forces exerted on chromosomes. Numbers in parentheses indicate the number of cells examined. *p < 0.001 (Student's *t*-test). Error bars indicate standard errors. (*h*) Distribution of angles of splitting sister centromeres. Dotted red lines show the distributions of wild-type and *rec12Δ* cells. (*i*) Increased tilting of splitting sister centromeres in *dam1Δ* cells. Green bars: 0° ≤ *θ* < 20°; brown bars: 20̊ ≤ *θ* < 60°; blue bars: 60° ≤ *θ* ≤ 90°. +, no other mutations. Eighty-three and 56 splitting sister centromeres were examined for *dam1Δ* and *dam1Δ rec12Δ* cells, respectively.

Importantly, the *dam1Δ* mutation impaired disjunction of homologous chromosomes (figure 5*a*), as seen in *mad2Δ* and *ark1-so* mutants [25,52]. Furthermore, the *dam1Δ* mutation increased equational segregation of sister chromatids in *rec12^+^* cells (figure 5*b*, left) but it decreased equational segregation in *sgo1Δ rec12Δ* or haploid meiotic *sgo1Δ* cells (figure 5*b*,*c*). Consistent with the equational segregation frequencies, the mean splitting frequencies of sister centromeres decreased in *dam1Δ rec12Δ* cells, and although the difference was not significant, the mean splitting frequencies tended to be higher in *dam1Δ* cells (figure 5*d*,*e*; electronic supplementary material, figure S2A). In addition, opposing forces exerted on homologous centromeres decreased, as demonstrated by the increase in tilted, non-parallel positioning of homologous centromeres and the reduction in their distances (figure 5*f*,*g*). These phenotypes mirror those of error correction-defective *mad2Δ* and *ark1-so* mutants (figure 3*b*,*c*). Furthermore, as seen in *ark1-so* cells, the proportion of splitting sister centromeres that were tilted towards a perpendicular direction increased irrespective of chiasma formation (figure 5*h,i*; electronic supplementary material, figure S3). These similarities to error correction-defective mutants strongly suggest that attachment correction is defective in *dam1Δ* cells. The *dam1Δ* mutation significantly shortened the mean projected distances (electronic supplementary material, figure S4C,D), suggesting a reduction in opposing forces exerted on sister centromeres.

The attachment changes did not result from premature anaphase onset or impaired Aurora B localization, as *dam1Δ* cells exhibited delayed anaphase onset (metaphase duration: 11.4 ± 0.7 min in wild-type cells (*n* = 10) and 31.5 ± 2.3 min in *dam1Δ* cells (*n* = 6)) and Aurora B localization patterns similar to those seen in wild-type cells (pre-anaphase punctate localization adjacent to kinetochores and anaphase spindle localization; electronic supplementary material, figure S7A,B).

### 2.5. Dam1 is required for metaphase centromere oscillation and anaphase A

**Figure 6.**
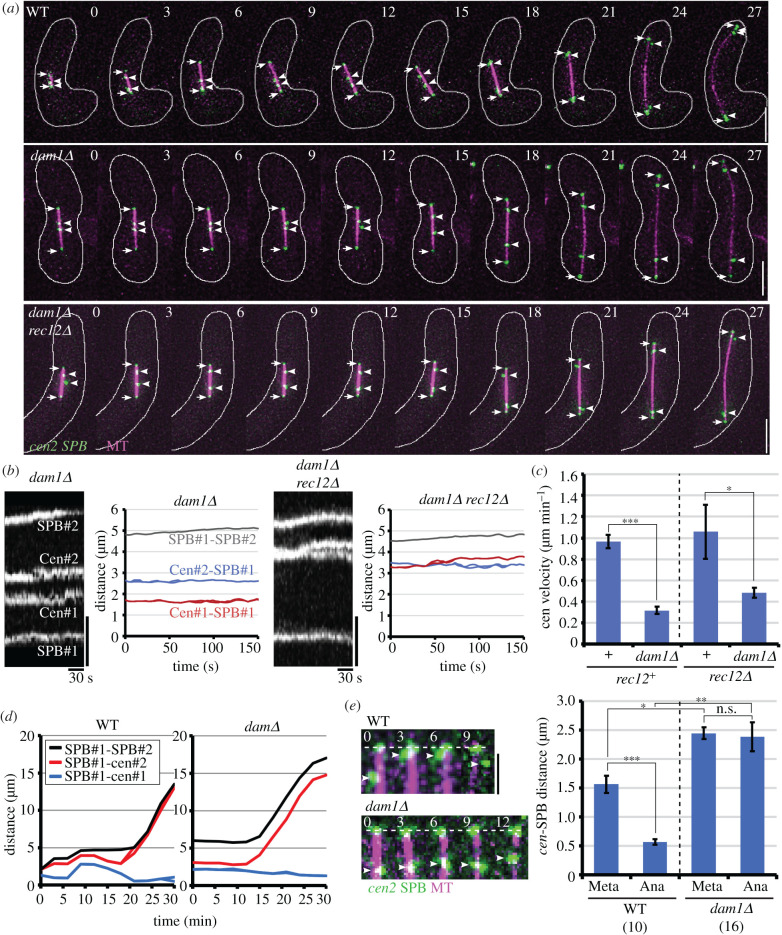
Centromere and spindle dynamics at meiosis I in *dam1Δ* cells. (*a*) Behaviour of centromeres (*cen2*) and the spindle at meiosis I. Green indicates *cen2* and SPB, and magenta indicates the spindle. Numbers indicate time (in minutes). Arrows and arrowheads indicate the SPB and *cen2*, respectively. White lines indicate cell outlines. Bar, 5 µm. (*b*) Changes in centromere and SPB positions at metaphase I in *dam1Δ* and *dam1Δ rec12Δ* cells. Left images show kymograph of centromeres (*cen2*) and SPBs and right graphs show changes in centromere–SPB and SPB–SPB distances. Vertical and horizontal bars of kymographs show 2 µm and 30 s, respectively. (*c*) Mean centromere velocities. Dotted lines distinguish results of *rec12^+^ and rec12Δ* cells. (*d*) Changes in centromere–SPB and SPB–SPB distances during anaphase I. (*e*) Loss of anaphase A centromere movements in *dam1Δ* cells. Photos show changes in centromere positions during anaphase I. Numbers show time in minutes from the start of anaphase. Dotted lines and arrowheads in photos show positions of SPB and *cen2*, respectively. Bar, 2 µm. Graph shows the mean centromere–SPB distances during metaphase I (Meta) and anaphase I (Ana). Numbers of centromeres examined are shown in parentheses. A dotted line in graph distinguishes results of wild-type and *dam1Δ* cells. Error bars show standard error. *p < 0.05; **p < 0.01; ***p < 0.0005; n.s., not significant (Student's *t*-test).

We also found that centromere oscillation was abolished in *dam1Δ* cells. At metaphase I in both *dam1Δ* and *dam1Δ rec12Δ* cells, spindle length tended to increase (electronic supplementary material, figure S8A), perhaps reflecting the loss of inward pulling forces generate by kMT depolymerization [53], and although centromeres were on the spindle, as in wild-type cells, they remained largely still without undergoing oscillation (figure 6*a*,*b*; electronic supplementary material, Movies S7–S11). Accordingly, centromere velocities and the standard deviation of centromere positions decreased markedly, and the MSD plots shifted downward (figure 6*c*; electronic supplementary material, figure S8B,C). The auto- and cross-correlation plots exhibited different patterns from those in wild-type and *rec12Δ* cells (electronic supplementary material, figure S8D–F).

In addition to centromere oscillation, anaphase poleward centromere movements were impaired in *dam1Δ* cells. Upon anaphase onset, homologous centromeres normally separate from each other by poleward movements (anaphase A), and then by spindle elongation (anaphase B) (figure 6*a*,*d*,*e*, WT) [23]. In *dam1Δ* cells, however, anaphase poleward centromere movements rarely occurred, and their distances from the nearest SPBs were largely unchanged (figure 6*a,d,e*; electronic supplementary material, figure S8G,H). Nonetheless, homologous centromeres successfully separated from each other as the spindle elongated (figure 6*a*,*d*), indicating that homologous centromeres undergo anaphase B without undergoing anaphase A. Notably in this regard, anaphase A movements were impaired in *rec12Δ* cells due to bi-oriented attachment of sister chromatids (electronic supplementary material, figure S8H) [28]. The lack of centromere oscillation and anaphase A are consistent with previously reported *dam1Δ* phenotypes: kinetochores remain at the MT ends for a long time (more than 20 min) and inhibit MT disassembly after kinetochore attachment to the MT ends [43,54], and chromosomes frequently fail to reach the SPB during mitotic anaphase [51,55]. The coincidental impairment of centromere oscillation, together with the attachment correction defect, provides further support for a link between attachment correction and centromere oscillation.

## 3. Discussion

### 3.1. Roles of chiasmata and Dam1 in attachment correction

In this study, we investigated whether chiasmata cause elimination of bi-oriented attachment via the error correction mechanism by analysing the attachments in *mad2Δ* or *ark1-so* cells forming or lacking chiasmata. Analysis of sister chromatid segregation, transient sister centromere splitting and homologous centromere positioning collectively revealed that in the *mad2Δ* and *ark1-so* backgrounds, bi-oriented attachment of sister chromatids increased in chiasma-forming cells, but mono-oriented attachment increased in chiasma-lacking cells. Presumably, sister chromatids are initially frequently bi-oriented irrespectively of chiasma formation, but the error correction mechanism eliminates bi-oriented attachment specifically in chiasma-forming cells. Less prominent impacts of the *mad2Δ* mutation on attachments than those of the *ark1-so* mutation probably resulted from distinct mutation-dependent defects. The *mad2Δ* mutation decreases the time available for correction but does not compromise attachment elimination unlike the *ark1-so* mutation; in *mad2Δ* cells, despite attachment elimination, the shortage of the correction time probably resulted in incomplete attachment correction. Together, these observations indicate that chiasmata enable error correction factors to eliminate bi-oriented attachment of sister chromatids that are otherwise not eliminated.

A previous study reported that *mad2* and *ark1* mutations increased bi-oriented attachment of sister chromatids in the absence of chiasmata [31], contradicting the results of this study. The *ark1-as* mutation and a different centromere marker used in that previous study may have affected attachments. In addition, it is important to note that pairing of homologous centromeres, which occurs during meiotic prophase, may affect the initial kinetochore–MT interaction. However, the contribution of centromere pairing to the observed differences between chiasma-forming and chiasma-lacking cells is probably negligible because centromere pairing occurs at a largely normal level in *rec12Δ* cells [56] and equational segregation frequencies in haploid meiotic cells, which lack pairing, were almost the same as those in *rec12Δ* cells (figure 1*e*,*f*).

In addition to bi-oriented attachment of sister chromatids, our analysis of sister centromere tilting suggested frequent occurrence of merotelic attachment and its elimination by the error correction mechanism, irrespective of chiasma formation. Indeed, merotelic attachment is frequently observed in chiasma-forming meiotic cells as well as in chiasma-lacking mitotic cells [57,58]. Tilted centromere positioning may have resulted from only one of the sister centromeres interacting with MT (monotelic attachment) or from detachment of sister centromeres from MTs after separation. However, given the active mobilities of splitting sister centromeres (electronic supplementary material, figure S2B–D) and the stable end-on attachment seen in *dam1Δ* cells [43,54], these events are likely to be rare. It is also possible that both sister centromeres interact with MTs extending from the same pole (syntelic attachment), one laterally and the other at the ends, as in vertebrate cells [59], and that bi-directional lateral sliding of one of the centromeres causes tilted positioning. However, the consistency of the frequencies of sister centromere splitting with those of equational segregation suggests that syntelic attachment is probably transient, if it occurs at all.

We also found that the *dam1Δ* mutation increased bi-oriented attachment of sister chromatids in chiasma-forming cells and increased mono-oriented attachment in chiasma-lacking cells. This suggests that Dam1 plays a crucial role in the attachment correction. The increased tilting of splitting sister centromeres seen in *rec12^+^ dam1Δ* cells further supports this notion. Because *dam1Δ* cells exhibited delayed anaphase onset, Dam1 must contribute to attachment elimination via an SAC-independent pathway. Given that Dam1 plays a crucial role in kinetochore–MT interaction and is a substrate of Aurora B [42,51,60], we speculate that the Dam1 complex is directly involved in the attachment elimination process as a component of the Aurora B regulatory pathway.

### 3.2. Functions of chiasmata and Dam1 in centromere oscillation

Using the correlation functions, we showed that centromeres oscillated irrespectively of chiasma formation and that chiasmata coordinated homologous centromere oscillation. Various experimental- and/or simulation-based studies demonstrated that coordinated oscillation of sister chromatids depends on the tension generated by sister centromere cohesion [10,61–71]. Given this notion, it is likely that coordinated homologous centromere oscillation similarly depends on tension generated by chiasmata. During oscillation of mitotic sister centromeres, kinetochore-interacting MT bundles (kMTs) extending forward of the moving centromeres (leading kMTs) drive centromere movements by their disassembly, whereas those extending rearward (trailing kMTs) assemble (electronic supplementary material, figure S9A, centromere movement and kMT dynamics) [4–6,10], and switching of either the leading or trailing kMTs induces reversal of centromere movements (electronic supplementary material, figure S9A, centromere movement and kMT dynamics) [72]. In fission yeast, Kinesin-8 motors, Klp5 and Klp6, that promote disassembly of longer kMTs induce kMT switching in the proximity of the equator [41,71,73,74]. The kMT switching causes transient centromere relaxation or stretching (electronic supplementary material, figure S9A, mitosis, transition state), which likely induces coordinated assembly/disassembly switching of the other kMTs through force-dependent changes in MT dynamics [75–78]. During homologous centromere oscillation, the kMTs probably undergo assembly and disassembly in a similar manner, and the chiasma-dependent link generates a temporal tensile change that coordinates assembly/disassembly switching of the kMTs (electronic supplementary material, figure S9A, meiosis I).

We also found that Dam1 was essential for centromere oscillation and anaphase A poleward centromere movements. This finding indicates that centromere oscillation and anaphase A depend on the same mechanism. The Dam1 complex probably drives centromere movements by coupling MT disassembly to centromere movement [49,50]. It may also promote MT disassembly, as kMT shortening is inhibited after kinetochore capture in *dam1Δ* cells [43,54,79]. Despite the lack of centromere oscillation and anaphase A, kinetochore–MT interactions were not eliminated in *dam1Δ* cells, as demonstrated by sister centromere splitting and anaphase B centromere movements. Therefore, a factor(s) in addition to Dam1 may cooperatively drive centromere movement. The factors that are likely to mediate kinetochore–MT interaction in *dam1Δ* cells include the Ndc80 complex, a distinct kinetochore–MT interface factor and the Klp5/Klp6 complex, which can couple MT disassembly to cargo movement like the Dam1 complex and play crucial roles in centromere oscillation and/or chromosome segregation [71,73,74,80–87]. Indeed, kinesin-8 motors are essential in *dam1Δ* cells [51] and contribute to stable kinetochore–MT interaction during centromere oscillation [73].

### 3.3. Functions of chiasma and Dam1 in attachment elimination

The precise mechanism of chiasma-dependent attachment elimination and the mechanism of Dam1-dependent attachment elimination itself remain unclear. It was previously proposed that chiasmata eliminate bi-oriented attachment of sister chromatids by generating a chromosome configuration that arranges sister kinetochores outward and the Aurora B-enriched region inward [31,88]. In this model, poleward pulling brings improper attachment sites close to the Aurora B-enriched region, leading to the elimination of the attachments. However, this configuration requires firm association of sister centromeres. When sister centromeres separate as seen in our study, improper attachment sites would barely approach the Aurora B-enriched region (electronic supplementary material, figure S9B). Therefore, the validity of this model remains debatable.

It is possible that chiasmata eliminate bi-oriented attachments by reducing tension across sister centromeres. Indeed, although we could not observe significant difference between projected inter-sister centromere distances of *rec12^+^* and *rec12Δ* cells, a subset of the distances in the *rec12^+^* cells covered a slightly shorter distance range than those covered in *rec12Δ* cells, suggesting a reduction in tension. However, in a *rec12^+^* cell shown in figure 2, the projected centromere distances were comparable to or greater than those in *rec12Δ* cells (electronic supplementary material, figure S4C), suggesting that at least in this particular cell, tension was probably not reduced. Nonetheless, the bi-oriented attachment was eliminated as demonstrated by re-association of the splitting sister centromeres (figure 2*b*,*c*, WT). Therefore, we speculate that chiasma-dependent attachment elimination is not solely dependent on the reduction in overall tension.

Our finding that both chiasmata and Dam1 contribute to centromere oscillation raises the possibility that attachment elimination is coupled to centromere oscillation. This possibility is supported by our finding that homologous centromere oscillation dynamics are altered in the error correction-defective *ark1-so* mutant. One possible explanation is that centromere oscillation causes elimination of erroneous attachments. In chiasma-forming cells, when improperly attached homologous centromeres undergo oscillation via coordinated disassembly and assembly of the leading and the trailing kMTs (electronic supplementary material, figure S9C, Meiosis I), stochastic and/or length-dependent initiation of assembly/disassembly MT switching may give assembling kMT ends a chance to experience a chiasma-dependent minus end-directed load at improper attachment sites. The minus end-directed load applied to the assembling kMT ends may bring attachment sites close to the inner centromere region, inducing Aurora kinase-dependent kMT detachment. Alternatively, the minus end-directed load may cause kMT detachment due to the intrinsically weak resistance of the interaction between assembling MTs and the kinetochore to a minus end-directed load. Indeed, attachment of Stu2, a XMAP215/Dis1 family kinetochore component in budding yeast, to assembling MT ends can withstand a tensile force of approximately 4 pN under plus end-directed load [89], whereas attachment of *Xenopus* XMAP215 can withstand a force of only approximately 1 pN force under minus end-directed load [90]. In this model, chromosome oscillation actively eliminates improper attachments by applying a load to the improper attachment sites, and the lack of chiasmata or centromere oscillation impairs attachment elimination.

An alternative, but not mutually exclusive, possibility is that attachment elimination requires an attachment property that enables centromere oscillation. We found that centromere oscillation is impaired in *ark1-so* cells. In addition, expression of mutant forms of the kinetochore component Ndc80 that cannot be phosphorylated by Aurora B inhibits centromere oscillation [81,83]. These observations indicate that centromere oscillation requires Aurora B-dependent kinetochore phosphorylation. In addition, variants of Dam1 or Ndc80 bearing Aurora B-phosphomimetic mutations form diffusible attachment to the MT lattice and exhibit diffusion-like movements on the MT [44,46,91]. Therefore, centromere oscillation probably depends on Aurora B-dependent diffusible attachment. Because diffusion activities of kinetochore components increase as their MT affinities decrease, only diffusible attachments may allow attachment elimination. In the absence of Dam1, attachment may become non-diffusible and non-eliminable, resulting in impairment of both centromere oscillation and elimination of erroneous attachments. Further close investigation of the effects of the phospho-mutant forms of Dam1/Ndc80 on attachment correction and centromere oscillation would be important to verify this possibility.

The chromosome oscillation-dependent mechanism can also account for the elimination of merotelic attachment of a single chromatid in a chiasma-independent manner and in both mitosis and meiosis. Perhaps, cohesin-dependent coordinated oscillation of sister chromatids eliminates merotelic attachment in the same way that coordinated homologous chromosome oscillation eliminates bi-oriented attachment of sister chromatids (electronic supplementary material, figure S9C, mitosis). Consistently, a loss of sister chromatid cohesion causes merotelic attachment in mitosis [92,93], and in *dam1Δ* cells, impaired centromere oscillation was accompanied by increased sister centromere tilting, a probable outcome of merotelic attachment, irrespectively of chiasma formation (figure 5*i*). Furthermore, the oscillation-dependent mechanisms likely contribute to attachment elimination in vertebrates, although the mechanisms are not completely the same, as demonstrated by additional contribution of Aurora A kinase to centromere oscillation and attachment correction [94,95].

The oscillation-dependent mechanism does not deny the importance of overall tension across sister centromeres in attachment elimination. It is clear that as the number of properly attached kMTs increases, tension across sister centromeres increases. Gradual elevation of tension may incrementally decrease kinetochore phosphorylation levels, resulting in an incremental increase in MT binding affinity of kinetochores, as in the case of Ndc80 phosphorylation [91]. An alternative, but not mutually exclusive, possibility is that tension greater than some threshold alters the kinetochore phosphorylation state. Kinetochore phosphorylation is regulated by phosphatases in addition to Aurora B [96], and the antagonistic actions of these enzymes may robustly maintain the kinetochore phosphorylation state during metaphase, as suggested for antagonistic regulatory systems [97]. However, once tension exceeds the threshold, the kinetochores may be completely dephosphorylated by the kinetochore regulatory system [96,98,99]. In either case, tension converts the metaphase-type diffusible, correctable attachments to the anaphase-type non-diffusible, non-correctable attachments, linking establishment of proper attachments with the metaphase-to-anaphase transition. This scenario is consistent with the well-established relationship between tension and loss of Aurora-dependent phosphorylation and attachment stabilization (e.g. [12,27,100, 101]). In addition, in this scenario, a reduction in tension leads to an increase in kinetochore phosphorylation, making the kinetochore state preferable for attachment correction; this can account for correction of merotelic attachment induced by reduced tension [102].

In this study, we showed that chiasmata and Dam1 differentially contribute to the elimination of erroneous attachments and centromere oscillation. These findings raise the possibility that attachment elimination is coupled with centromere oscillation. The precise mechanisms of chiasma-dependent or Dam1-dependent attachment elimination, as well as the relationship between centromere oscillation with attachment elimination, remain to be elucidated. However, our findings and the suggested relationship will undoubtedly shed new light on understanding of the mechanisms of attachment correction and centromere oscillation. Because chromosome missegregation is associated with various diseases or disorders, including tumorigenesis and birth-related Down's syndrome, our findings may be of clinical importance.

## 4. Material and methods

### 4.1. Yeast strains, media and basic genetical methods

Strains used in this study are shown in electronic supplementary material, tables S2 and S3. Media and basic genetical methods used in this study were described previously [103]. The *dam1* deletion strain was generated by replacing the entire *dam1* gene with the G418-resistance gene by a PCR-based method [104,105].

### 4.2. Analysis of sister chromatid segregation at meiosis I

Sister chromatid segregation at meiosis I in diploid zygotes and haploid meiotic cells was examined as previously described [28].

### 4.3. Visualization of Cut2

To visualize Cut2, plasmid pKK13 encoding mCherry-tagged Cut2 was constructed as follows. A DNA fragment containing the promoter and ORF of the *cut2* gene was amplified by PCR using the oligonucleotide primers 5′-ACGCGTCGACATGCGACGTTTGTTGTGCCC-3′ and 5′-CGGGATCCCTAACAATCCTGTATCCAAAGATGA-3′, with genomic DNA as the template. The amplicon was inserted between *Sal*I and *BamH*I sites of the *mCherry*-bearing plasmid pHM4 [106], yielding pKK13. Strains expressing mCherry-tagged Cut2 were constructed by introducing pKK13 into cells.

### 4.4. Time-lapse analysis of centromere and SPB positions at meiosis I in live cells

Cells of opposite mating types containing the GFP-visualized *cen2* locus were grown on YES solid medium at 30°C and mixed on ME solid medium. They were induced to enter meiosis by incubation at 25°C for 16–18 h. The cells were suspended in EMM-N liquid medium, and a drop of the suspension was placed on the bottom of 35 mm glass-bottom dishes (Matsunami Glass Ind., Ltd, Osaka, Japan) coated with 5 mg ml^−1^ lectin (Sigma-Aldrich Japan, Tokyo, Japan). Metaphase I zygotes containing Cut2-mCherry signals were chosen, and time-lapse images of centromeres and SPBs in these cells were collected at seven focal planes spaced at 0.5 µm intervals once every approximately 3 s on an IX71 inverted microscope equipped with a cooled charge-coupled device camera (CoolSNAP-HQ2; Nippon Roper Co. Ltd, Tokyo, Japan) and a 100× /1.40 NA Plan Apo oil-immersion objective lens (Olympus, Tokyo, Japan). During observation, the zygotes were kept at 25°C. The resultant images were processed by deconvolution and analysed using the MetaMorph (version 7) (Molecular Devices Japan, Tokyo, Japan) or Priism/IVE software [107]. Three-dimensional coordinates of GFP-visualized centromeres and SPBs were determined using a multi-dimensional motion analysis module in the MetaMorph software.

For acquisition of time-lapse images of GFP-visualized centromeres and SPBs and RFP-visualized spindles, cells were observed on a DeltaVision microscope system equipped with a 60 × /1.42 NA Plan Apo oil-immersion objective lens (Olympus) operated by SoftWoRx software (Applied Precision Inc.). Time-lapse images of cells were collected at 10 focal planes spaced at 0.4 µm intervals every 3 min using a cooled CCD camera. During collection of time-lapse images, the cells were kept at 25°C in a microscope chamber. The resultant images were processed by deconvolution using SoftWoRx and analysed using the Priism/IVE software [107].

### 4.5. Analysis of centromere movements

To characterize centromere movements, the distance between the centromere and the nearest SPB at each time point was measured, and moving averages of centromere–SPB distances over a window of three consecutive time points were determined. When sister centromeres were separated, a centroid of the two sister centromere coordinates was used. Because time intervals often varied due to differences in the amounts of time required for image acquisition, centromere–SPB distances separated by constant time intervals were calculated from the moving averages of the centromere–SPB distances, assuming that the centromeres moved at a constant velocity between each pair of consecutive time points. Centromere velocities relative to the SPB were then determined by linear regression analysis, using the calculated distances separated by constant time intervals over a three-time point sliding window. The calculated centromere velocities obtained from all observed cells of each strain were collected and used for auto- and cross-correlation analyses.

The correlation functions were obtained as statistical averages over time and cells. Let *N* be the number of cells contained in the experimental dataset obtained for a specific genotype, and let $v_{1}^{\left(n\right)}(i)$ and $v_{2}^{\left(n\right)}(i)$ denote the velocities of the two centromeres in the nth cell at time iΔt, where n=1,2, …, N labels the individual cells contained in the dataset, *i* is an integer and Δt is the time interval. Specific values of Δ*t* were 3.1 s for the WT strain, 3.2 s for *ark1-so* and *ark1-so rec12Δ* strains, 3.3 s for *mad2Δ*, *dam1Δ* and *dam1Δ rec12Δ* strains, and 3.4 s for *rec12Δ* and *mad2Δ rec12Δ* strains. The sample averages of the products of two velocities at different times spaced by jΔt were calculated using the following equations:

4.1 $$\left\langle v(0)v(j)\right\rangle =\frac{\sum_{n=1}^{N}\sum_{i}^{\prime }\{v_{1}^{\left(n\right)}(i)v_{1}^{\left(n\right)}(i+j)+v_{2}^{\left(n\right)}(i)v_{2}^{\left(n\right)}(i+j)\}}{\sum_{n=1}^{N}\sum_{i}^{\prime }2}$$

and

4.2 $$\left\langle v(0)v^{\prime }(j)\right\rangle =\frac{\sum_{n=1}^{N}\sum_{i}^{\prime }\{v_{1}^{\left(n\right)}(i)v_{2}^{\left(n\right)}(i+j)+v_{2}^{\left(n\right)}(i)v_{1}^{\left(n\right)}(i+j)\}}{\sum_{n=1}^{N}\sum_{i}^{\prime }2},$$

where the prime over *v* on the left-hand side of equation (4.2) signifies that the product is taken of the velocities of the different centromeres. The prime over Σ in equations (4.1) and (4.2) signifies that the summation over *i* is taken over the values of *i* for which the data of both $v_{k}^{\left(n\right)}(i)$ and $v_{k}^{\left(n\right)}(i+j)$ (k=1,2) exist. The range of *i* for which the measured data exist corresponds to the time interval during which the cell was found in the focal region. The denominators in equations (4.1) and (4.2) count the number of data points included in the summation in the numerators.

The velocity auto-correlation function (ACF) and cross-correlation function (CCF) at delay jΔt were then estimated by

4.3 $$\mathrm{ACF}(\;j\Delta t)=\frac{\left\langle v(0)v(j)\right\rangle }{\left\langle v(0)v(0)\right\rangle }$$

and

4.4 $$\mathrm{CCF}(\;j\Delta t)=\frac{\left\langle v(0)v^{\prime }(j)\right\rangle }{\left\langle v(0)v(0)\right\rangle }.$$

### 4.6. Determination of confidence intervals by bootstrap analysis

The confidence intervals for centromere split frequencies, average split distances and velocity correlation functions measured in this study were evaluated by the basic bootstrap method [108]. In bootstrap methods, we perform re-sampling of the samples of *N* cells, which we denote by $\left\{n_{1}^{*},n_{2}^{*},\ldots ,n_{N}^{*}\right\}$, where the numbers $n_{\ell }^{*}\;(\ell =1,2,\ldots ,\;N)$ are randomly sampled from {1,2,…, N} with replacement. The estimation of a target quantity *T* performed for this re-sampled dataset yields a re-sampled estimate $T^{*}$, where the letter *T* is used to denote either the split frequency, the average split distance or the velocity correlation of a particular strain. For example, the velocity correlation functions by this re-sampled set are given, corresponding to equations (4.1)–(4.4), by

4.5 $${\left\langle v(0)v(j)\right\rangle }^{*}=\frac{\sum_{\ell =1}^{N}\sum_{i}^{\prime }\{v_{1}^{\left(n_{\ell }^{*}\right)}(i)v_{1}^{\left(n_{\ell }^{*}\right)}(i+j)+v_{2}^{\left(n_{\ell }^{*}\right)}(i)v_{2}^{\left(n_{\ell }^{*}\right)}(i+j)\}}{\sum_{\ell =1}^{N}\sum_{i}^{\prime }2},$$

4.6 $${\left\langle v(0)v^{\prime }(j)\right\rangle }^{*}=\frac{\sum_{\ell =1}^{N}\sum_{i}^{\prime }\{v_{1}^{\left(n_{\ell }^{*}\right)}(i)v_{2}^{\left(n_{\ell }^{*}\right)}(i+j)+v_{2}^{\left(n_{\ell }^{*}\right)}(i)v_{1}^{\left(n_{\ell }^{*}\right)}(i+j)\}}{\sum_{\ell =1}^{N}\sum_{i}^{\prime }2},$$

4.7 $$\mathrm{ACF}{\left(\;j\Delta t\right)}^{*}=\frac{{\left\langle v(0)v(j)\right\rangle }^{*}}{{\left\langle v(0)v(0)\right\rangle }^{*}}$$

4.8 $$\mathrm{and}\;\mathrm{CCF}{\left(\;j\Delta t\right)}^{*}=\frac{{\left\langle v(0)v^{\prime }(j)\right\rangle }^{*}}{{\left\langle v(0)v(0)\right\rangle }^{*}},$$

where ∗ denotes the estimates made using the re-sampled data set. The re-sampling is then repeated $R_{1}$ times, with $R_{1}$ being a sufficiently large number. We then obtain a set of $R_{1}$ values of $T^{*}$, giving a distribution of $T^{*}$.

Let $a_{q}^{*}$ be the value that makes the probability that $T^{*}\leq T+a_{q}^{*}$ equal to *q*. Then, the confidence region for the true value θ of *T* with confidence level α (where we take 1−2α=0.95) is given by

4.9 $$t-a_{1-\alpha }^{*}<\theta <t-a_{\alpha }^{*},$$

where *t* is the value of *T* obtained using the original data set. To estimate $a_{q}^{*}$, we use the $R_{1}$ re-sampled values $t_{1}^{*},\;t_{2}^{*},\;\ldots ,\;t_{R_{1}}^{*}$ of $T^{*}$, which we re-order as $t_{\left(1\right)}^{*}\leq t_{\left(2\right)}^{*}\leq \cdots \leq t_{\left(R_{1}\right)}^{*}$. The estimate for $a_{q}^{*}$ is then given by $a_{q}^{*}=t_{\left((R_{1}+1)q\right)}^{*}-t$. In practice, we took $R_{1}=5000$ for the number of re-samplings.

For the evaluation of the confidence interval for the velocity correlation, we first performed the transformation to the variance-stabilized scale

4.10 $$T=\frac{1}{2}\log\left(\frac{1+C}{1-C}\right),$$

where we have either C=ACF( jΔt) or C=CCF( jΔt) for a particular value of *j*. The variable *T* has a one-to-one correspondence with *C*, and under normal approximation, its variance is independent of the true value of *C* [108]. The confidence interval of this *T* was evaluated by the method explained above, and the backward transformation of equation (4.10) was performed to give the confidence interval of *C*.

### 4.7. Statistical significance test by bootstrap analysis

The difference in centromere split frequencies, average split distances and velocity correlation functions between two strains was tested for statistical significance by the basic bootstrap method. Again, let the letter *T* denote either the split frequency, the average split distance or the velocity correlation of a particular strain. In the cases of the split frequency and the split distance, the quantity is given by a simple weighted average of all the cells contained in the strain. For strain *k* containing $N_{k}$ cells

4.11 $$T_{k}=\frac{\sum_{n=1}^{N_{k}}w_{n}x_{n}}{\sum_{n=1}^{N_{k}}w_{n}},$$

where $w_{n}$ is the number of observed time points in the nth cell and $x_{n}$ is the split frequency or the average split distance in the nth cell.

We wish to compare the values $T_{1}$ and $T_{2}$ for two strains, named 1 and 2 here. With the sample variance $S_{k}^{2}$ given by

4.12 $$S_{k}^{2}=\frac{\sum_{n=1}^{N_{k}}w_{n}x_{n}^{2}\;}{\sum_{n=1}^{N_{k}}w_{n}}-T_{k}^{2},$$

we consider the following quantity [108], which is similar to that used in the usual *t*-test:

4.13 $$Z=\frac{T_{1}-T_{2}}{\sqrt{S_{1}^{2}/(N_{1}-1)+S_{2}^{2}/(N_{2}-1)}}.$$

Bootstrap re-sampling was performed by randomly sampling $\left\{n_{11}^{*},\;n_{12}^{*},\ldots ,\;n_{1N_{1}}^{*}\right\}$ from $\left\{1,2,\;\ldots ,\;N_{1}\right\}$ with replacement for strain 1 and $\left\{n_{21}^{*},\;n_{22}^{*},\;\ldots ,\;n_{2N_{2}}^{*}\right\}$ from $\left\{1,2,\ldots ,N_{2}\right\}$ for strain 2. The re-sampled statistic

4.14 $$Z^{*}=\frac{\left(T_{1}^{*}-T_{2}^{*})-(T_{1}-T_{2}\right)}{\sqrt{S_{1}^{*2}/(N_{1}-1)+S_{2}^{*2}/(N_{2}-1)}},$$

was calculated for each of $R_{1}$ re-samples. Then the p-value was evaluated by

4.15 $$p=\{\begin{array}{c} \frac{\#\{Z^{*}>Z\}+1}{R_{1}+1},\;\mathrm{if}\;\;Z>0\;\\ \frac{\#\{Z^{*}<Z\}+1}{R_{1}+1},\;\mathrm{if}\;\;Z<0\;\; \end{array}$$

where $\#\{Z^{*}>Z\}$ means the number out of $R_{1}$ re-samples in which the value of $Z^{*}$ exceeded *Z*.

 In the case of correlation, which is not a simple average but the ratio of covariance and variance in equations (4.3) and (4.4), the method of pooled empirical distribution was used [108]. The $N_{1}+N_{2}$ cells were taken together to form a single set. The bootstrap re-sampling was performed by randomly sampling $\left\{n_{11}^{*},\;n_{12}^{*},\;\ldots ,\;n_{1N_{1}}^{*}\right\}$ from $\left\{1,\;2,\;\ldots ,\;N_{1}+N_{2}\right\}$ with replacement mimicking strain 1 and $\left\{n_{21}^{*},\;n_{22}^{*},\;\ldots ,\;n_{2N_{2}}^{*}\right\}$ from $\left\{1,\;2,\;\ldots ,\;N_{1}+N_{2}\right\}$ mimicking strain 2. Then, we counted the number of re-samples out of $R_{1}$ in which $\left|T_{1}^{*}-T_{2}^{*}\right|$ exceeded $\left|T_{1}-T_{2}\right|$. The count was then converted to the p-value similarly to equation (4.15).

### 4.8. Analysis of spore viability and meiotic recombination

For analysing spore viability and meiotic recombination, cells were grown on YES solid medium at 32°C and induced to enter meiosis on ME solid medium at 25°C. For assessing spore viability, spores in tetrads were dissected and placed on YES solid medium using a microneedle, and their viability was determined by colony formation. Crossover recombination between the *leu1* and the *his2* loci was analysed by tetrad analysis. Gene conversion at the *ade6* locus was examined by crossing cells harbouring the *ade6-M26* or the *ade6-M375* mutation and those harbouring the *ade6-L469* mutation. Spores were liberated from asci by incubating the asci in 0.5% β-glucuronidase (Fujifilm Wako Pure Chemical Corp., Osaka, Japan) at 30°C. Then spore suspensions were spread on YES and EMM plates, and the plates were incubated at 32°C. The frequencies of *ade^+^* colonies were determined by the number of colonies on the YES and EMM plates.

## Data accessibility

This article has no additional data.
